# A Case of Bouveret’s Syndrome Treated With Holmium:Yttrium-Aluminum-Garnet Laser

**DOI:** 10.7759/cureus.37258

**Published:** 2023-04-07

**Authors:** Aunchalee Jaroenlapnopparat, Aram N Demirjian, William R Brugge, Kinnari R Kher

**Affiliations:** 1 Internal Medicine, Mount Auburn Hospital, Harvard Medical School, Cambridge, USA; 2 Surgery, Mount Auburn Hospital, Harvard Medical School, Cambridge, USA; 3 Gastroenterology, Mount Auburn Hospital, Harvard Medical School, Cambridge, USA

**Keywords:** holmium:yag laser, endoscopy, gastric outlet obstruction, gallstone ileus, bouveret’s syndrome

## Abstract

Bouveret’s syndrome is a rare form of gallstone ileus described as a gastric outlet obstruction from a gallstone that travels from the gallbladder to the bowel through a bilioenteric fistula. Despite its rarity, the mortality rate of this condition is high. Endoscopic treatment is preferred over surgery due to the association with lower mortality rate. To date, there are limited data about the application of holmium:yttrium-aluminum-garnet (YAG) laser lithotripsy for fragmentation of gallstones in Bouveret's syndrome. We present the case of a 74-year-old man with multiple cardiac comorbidities who presented with periumbilical pain, decreased appetite, and vomiting. The patient had previously been admitted three months prior with acute cholecystitis, and a cholecystostomy tube was placed. He had leukocytosis and purulent discharge in his cholecystostomy bag. Computed tomography (CT) scan of the abdomen and pelvis showed a change in the position of a previously seen large gallstone from the neck of the gallbladder on the last admission, to the lumen of the duodenal bulb on this admission. This indicated the development of a cholecystoduodenal fistula, with the stone passing through this fistula into the duodenal bulb, causing the complete obstruction. Endoscopic treatment was recommended by the surgery team due to cardiac comorbidities and the significant friability of the tissue requiring laparotomy. Initial endoscopic evaluation showed complete obstruction of the duodenal bulb by a large smooth stone, not allowing passage of a guidewire beyond the stone. Therefore, holmium:YAG laser lithotripsy was used. After two sessions of laser therapy, four days apart, each breaking a pigmented and calcified stone, it eventually passed through the small bowel into the colon, relieving the obstruction. The patient had a favorable outcome and did not require surgery. This case report shows that holmium:YAG laser lithotripsy is capable of delivering favorable outcomes, as seen in a patient with a heavily calcified and pigmented stone, older age, and multiple comorbidities. Holmium:YAG laser could be considered for use with endoscopic equipment for future management of this condition, especially in patients who have medical comorbidities and heavily calcified gallstones.

## Introduction

About 10% of the general adult population in the United States has gallstone disease. Of these, 20-30% develop biliary pain or other complications from gallstones such as cholecystitis, cholangitis, pancreatitis, and gallstone ileus. Gallstone ileus is a condition when gallstones dislodge from the biliary system into the bowel lumen and cause bowel obstruction [1]. It is a rare complication of cholelithiasis, found in only 0.3-0.5% of patients with gallstones, and responsible for 1-4% cause of mechanical bowel obstruction cases [1]. Bouveret’s syndrome is a rare form of gallstone ileus described as a gastric outlet obstruction from a gallstone that travels from the gallbladder to the bowel through a bilioenteric fistula. Despite the rarity, the mortality rate of Bouveret’s syndrome used to be as high as 30%. Due to improvements in diagnostic and therapeutic methods, the recent mortality rate was reported to be around 12% [2]. This was mostly due to endoscopic and radiologic modalities being used, with improved mortality rates compared to surgery. However, data about the treatment of Bouveret’s syndrome, especially holmium:yttrium-aluminum-garnet (YAG) laser lithotripsy, remains limited. In this article, we present the case of an elderly man who presented with Bouveret’s syndrome and was managed with holmium:YAG laser lithotripsy.

This article was previously presented as a meeting abstract at the American College of Gastroenterology (ACG) 2022 conference on October 24, 2022.

## Case presentation

A 74-year-old male patient presented to the emergency department with two episodes of vomiting and one day of decreased appetite. He also reported periumbilical pain and nausea but no heartburn, fever, chills, or jaundice. His past medical history was significant for multiple cardiac comorbidities, including cardiomyopathy (left ventricular ejection fraction of 35%), atrial fibrillation, and ventricular tachycardia. He had an automatic implantable cardioverter defibrillator in place.

He had been admitted to our hospital three months prior with acute cholecystitis and elevated liver enzymes. Due to his cardiac comorbidities, he was treated with intravenous (IV) piperacillin and tazobactam 4.5 gm every six hours for five days, cholecystostomy tube, and endoscopic retrograde cholangiopancreatography (ERCP) with sphincterotomy. The liver enzymes improved, and he was discharged. Six weeks after discharge, a tube check had confirmed the correct placement of the tube and decompression of the gallbladder.

During this presentation, his pain was different from the right upper abdominal pain of his prior admission. Physical examination on this admission revealed epigastric and periumbilical tenderness with no guarding or rebound tenderness. His cholecystostomy bag had purulent drainage. His tube drainage had reduced considerably in the previous day.

**Figure 1:**
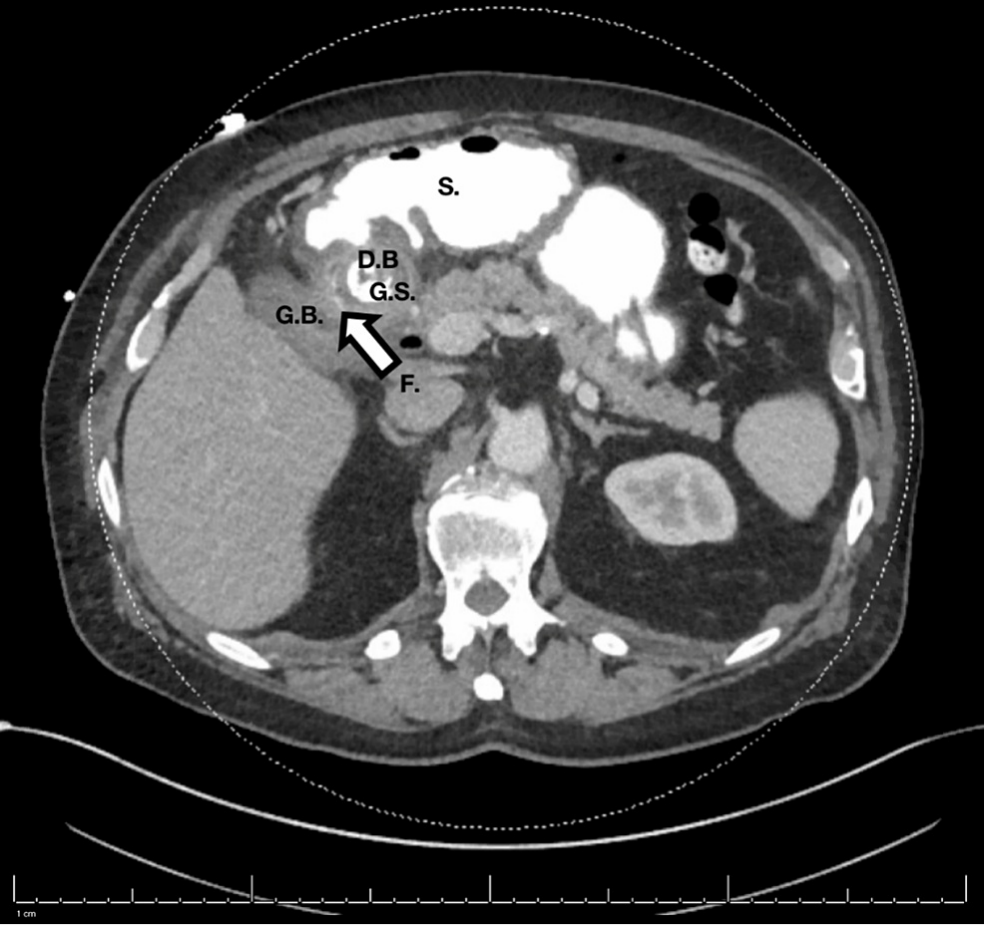
Computed tomography (CT) scan of the abdomen. A large gallstone (G.S.) was seen in the duodenal bulb (D.B) lumen adjacent to the gallbladder (G.B.), indicating a cholecystoduodenal fistula (F., arrow). Anterior to the duodenum is the stomach (S.), which is filled with contrast.

Laboratory results were significant for leukocytosis. He had normal liver enzymes and serum lipase. Computed tomography (CT) scan of the abdomen and pelvis showed a change in the position of a previously seen large 3.3 cm gallstone. During the previous admission, it was in the neck of the gall bladder. At this time, it was occupying the lumen of the duodenal bulb. This indicated the development of a cholecystoduodenal fistula, with the stone passing through this fistula into the duodenal bulb, causing complete gastric outlet obstruction (Figure 1). The patient received a seven-day course of IV ceftriaxone 1 g every 24 hours and IV metronidazole 500 mg every eight hours. The surgical team recommended endoscopic treatment for a less invasive approach due to the patient’s multiple medical comorbidities and the significant friability of the tissue in the setting of an acute fistula. This would make laparoscopic dissection difficult and increase the risk of requiring a laparotomy, which would increase the operative risk in a patient with multiple comorbidities.

**Figure 2:**
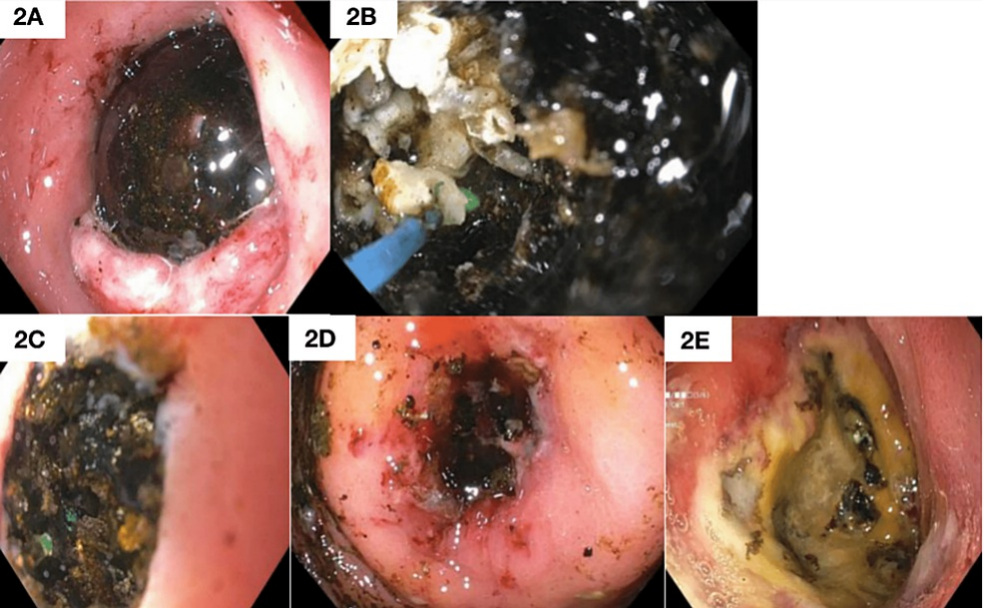
Endoscopic treatment of Bouveret's syndrome. (2A) A large gallstone seen at upper endoscopy, (2B) and (2C) application of holmium:YAG laser lithotripsy to the calcified and pigmented gallstone, (2D) despite the reduction in the gallstone size after the first lithotripsy session, the stone was not small enough to be retrieved through the pylorus, and (2E) residual ulcer in the duodenal bulb after the passage of the gallstone. YAG: yttrium-aluminum-garnet.

The initial endoscopic evaluation showed complete obstruction of the duodenal bulb by a large smooth stone, not allowing passage of even a guidewire beyond the stone (Figure 2). Following a multidisciplinary discussion, laser lithotripsy was selected to relieve the obstruction. A 410-micron holmium:YAG laser probe (Boston Scientific, Yokneam, Israel), which was more commonly used in urologic surgery, was used with an antegrade approach from the pylorus through a gastroscope in collaboration with the urology team. After two sessions of holmium:YAG laser lithotripsy, four days apart, each breaking a pigmented and calcified stone, it eventually passed through the small bowel into the colon, relieving the obstruction. The patient did not require surgical intervention, recovered well, and was discharged home. 

## Discussion

The level of obstruction in gallstone ileus is most commonly at the terminal ileum (60-70%), which is the narrowest part of the bowel, followed by the proximal ileum (25%), and then other parts of the small bowel. In rare cases of gallstone ileus (1-3%), the level of stone impaction can be located more proximally in the duodenum and cause gastric outlet obstruction. This rare condition is called Bouveret’s syndrome [3]. This syndrome was named after Leon August Bouveret, a French physician who described the condition in 1896. After an inflammatory process, such as acute cholecystitis, chronic inflammation from impacted gallstones can increase intraluminal pressure in the gallbladder resulting in wall ischemia and perforation and creating bilioenteric fistulas. The most common location of fistulas is cholecystoduodenal fistulas (68%), followed by cholecystocolic fistulas (17%), and the rarest is cholecystogastric fistulas (5%) [1]. Gallstones can move past the bilioenteric fistula and obstruct parts of the bowel, such as the duodenal bulb, causing obstruction of the gastric outlet. Beside an inflammatory process such as acute cholecystitis, gallbladder carcinoma can also cause bilioenteric fistulas [4]. In our case, the patient had previous acute cholecystitis and required a percutaneous cholecystostomy tube due to his multiple underlying diseases. The ongoing inflammation in his gallbladder from cholecystitis and the pressure of his large gallstone on to the gallbladder and bowel wall resulted in a cholecystoduodenal fistula. He then developed a gastric outlet obstruction at the level of the duodenal bulb.

The prevalence of this disease is highest in elderly females with a median age of 74 and a female-to-male ratio of 1.9 [1]. Risk factors include gallstones that are larger than 2.5 cm, recurrent biliary colic and chronic cholecystitis, and alteration of gastrointestinal anatomy after surgery [5]. The diagnosis of Bouveret’s syndrome can be challenging since the presenting symptoms are usually non-specific. Symptoms of gastric outlet obstruction can occur a week before the presentation. In the elderly with gastric outlet obstruction, a thorough investigation must be performed to rule out other etiologies, such as malignancy. Although Bouveret’s syndrome is a very rare disease, it comes with high mortality and physicians should be aware of not missing the diagnosis. A combination of imaging studies and clinical presentation are crucial to reach the diagnosis in time. Patients are most commonly present with symptoms such as nausea/vomiting (86%) and abdominal discomfort (71%). Patients can present with upper gastrointestinal bleeding (15%), weight loss (14%), and anorexia (13%). Physical examination usually reveals abdominal tenderness (44%), dehydration (31%), and abdominal distention (27%) [6]. In our case, the patient presented with periumbilical pain, nausea, vomiting, and a sudden reduction in the drainage from the cholecystostomy tube. These symptoms suggested gastric outlet obstruction and inflammation of the upper small bowel. 

History and physical examination alone are not adequate to make a diagnosis of Bouveret’s syndrome. Moreover, laboratory results are mostly non-specific. Imaging modalities such as abdominal plain film, ultrasound, and CT help identify the locations of stones and even the locations of fistulas. The most common initial imaging in the elderly who present with symptoms of bowel obstruction is the abdominal plain film. However, abdominal plain films are diagnostic in only 21% of Bouveret syndrome cases. Rigler’s triad, consisting of small bowel obstruction, pneumobilia, and ectopic gallstone, is a pathognomonic sign of gallstone ileus. However, this triad is detected in only 30-35% of patients by plain films. Hence, the abdominal plain film alone may not be enough to reach the diagnosis. An ultrasound of the abdomen may be useful if it shows ectopic gallstones and pneumobilia. However, ultrasound depends on the radiologist's skills and may not reveal the exact location of the stones. Moreover, pain on examination and excessive gas in the abdominal cavity are one of the limitations. CT abdomen is required in most cases and provides the best sensitivity (93%), specificity (100%), and accuracy (99%). CT abdomen can detect Rigler’s triad in up to 78% of cases [1]. Despite the excellent performance, the limiting factors of the CT scan are that 15-25% of gallstones can be isoattenuating, and this option might be limited for patients with renal failure. In case the abdominal CT does not reveal the stones, magnetic resonance cholangiopancreatography (MRCP) or esophagogastroduodenoscopy (EGD) are the next options to identify the locations of the stones. MRCP can detect Rigler’s triad in almost 100% of cases [1]. Interestingly, only 67% of stones and 13% of fistulas can be detected by EGD. As high as 20-40% of cases require surgery to reach the diagnosis [7]. In our case, the patient obtained a diagnosis after he underwent a CT abdomen with contrast, which revealed a large calcified gallstone in the duodenal bulb and a cholecystoduodenal fistula.

Even though the success rate of endoscopic treatment is less than 10%, endoscopy should be considered before surgery [8]. This is generally because of the higher mortality rate from surgery compared to endoscopy (17.3% vs. 1.6%, p=0.003) [9]. In some cases with younger age, less severe inflammation, and acceptable comorbidities, surgery may be considered over endoscopic treatment. Our patient was seen by the surgery team, which appropriately recommended an endoscopic approach due to the patient’s friable gallbladder and bowel mucosa, as well as his cardiac comorbidities. Ong and colleagues conducted a systematic review to identify factors that predict the outcomes of endoscopic therapy [10]. They reported that factors that are associated with successful endoscopic treatment in Bouveret’s syndrome are: gallstones with a diameter of 4 cm or less, impaction of gallstones in the proximal half of the duodenum, and multiple endoscopic modalities such as mechanical removal together with laser lithotripsy. Regarding endoscopic treatment, modalities such as nasogastric tube insertion to help decompress the stomach and esophageal overtube placement should be considered before pursuing any endoscopic treatment to improve outcomes. Endoscopic removal with a net or basket is a simple and quick method to relieve the gut obstruction. However, this method is generally used for small stones only [11]. Direct endoscopic removal can increase the risk of a stone being impacted in the esophagus. Moreover, distal obstruction of gallstone fragments can lead to gallstone ileus. Larger stones, especially when the size is more than 2.5 cm, may require additional methods to crush the stones into smaller pieces before endoscopic removal.

One of the techniques to crush gallstones into small fragments is mechanical lithotripsy. This is accomplished by using baskets, forceps, lithotripters, or snares to crush the stones into fragments, followed by endoscopic removal of stone fragments [12]. In cases where mechanical lithotripsy fails, laser lithotripsy, electrohydraulic lithotripsy (EHL), and extracorporeal shockwave lithotripsy (ESWL) can be considered. However, some of these methods may not be available in small centers. Laser lithotripsy is one of the most promising methods, using the mechanism of superheating the surrounding water and forming vaporizing bubbles to create a drilling effect from the heat. A variety of lasers include holmium:YAG, rhodamine, and neodymium (Nd):YAG. Laser lithotripsy provides the benefit of targeting the energy to the stones precisely, resulting in minimal damage to the surrounding tissue [11]. Holmium: YAG laser is commonly used in urosurgery to fragment urinary stones. In 2005, Goldstein et al. reported the first study that showed a successful outcome after extracting biliary stones by using holmium:YAG laser [13]. Later, a systematic review of 29 gallstones by Blomley and colleagues supported the efficacy of using holmium:YAG laser for gallstone lithotripsy [14]. From all available data, the holmium:YAG laser provides the best ability to apply high energy through small and flexible probes and should be considered as a first-line modality for gallstone lithotripsy [15]. There was a case report using a rhodamine laser to treat Bouveret’s syndrome successfully. However, it still required multiple attempts [16]. Nd:YAG laser is cheaper than holmium:YAG but it could not fragment all stone compositions [17]. Our patient underwent a holmium:YAG laser with a good outcome over two endoscopic sessions, despite having a large heavily calcified stone. EHL is more readily available in small centers and cheaper than laser lithotripsy. However, EHL increases shock wave dispersion, leading to a higher risk of surrounding tissue damage and can result in gastrointestinal bleeding and perforation [18]. The extracorporeal technique of ESWL has also been reported. However, the lack of endoscopic control is a big limitation. Despite being reported to be unsuccessful in most cases, ESWL might be a fine option when stones are not easily visualized. After extracting stones by endoscopy, patients may develop gastrointestinal ulcers at the areas where the stones compressed the wall. Close follow-up and a proton pump inhibitor should be considered to minimize complications such as gastrointestinal hemorrhage.

Even though there are multiple endoscopic modalities available, about 91% of the patients still have to undergo surgery [10]. Endoscopic treatment has some major limitations, such as endoscopist expertise. Also, only two-thirds of stones can be accessed by EGD. However, the low success rate of endoscopic methods does not necessarily mean that it is not a good option for treatment. Most patients with Bouveret’s syndrome are elderly and have multiple comorbidities, and diagnosis is often delayed. When surgical treatment is considered, a multidisciplinary collaboration between gastroenterology, surgery, radiology, anesthesiology, and others is necessary, given that management can be complex and require expert opinions. Open or laparoscopic approach, gastrotomy or duodenotomy, endoscopic assisted or not, are examples of the diversity of treatment options. Simultaneous cholecystectomy and fistula repair or "one-stage surgery" is still a debatable topic. Patients with cholecystoenteric fistulas have as high as a 17% chance of developing complications, such as cholecystitis, cholangitis, and recurrent gallstone. The risk of developing cholangiocarcinoma can increase 15-fold. On the contrary, a retrospective review of the United States Nationwide Inpatient Sample reported lower mortality in patients who underwent surgery for stone extraction alone compared to enterotomy and cholecystectomy with fistula closure [2]. Furthermore, a review of 1,001 cases by Reisner and colleagues reported that only 10% of gallstone ileus patients required reoperation for fistula repair [19]. Gallbladder wall tissue tends to be friable under chronic inflammatory processes such as Bouveret syndrome, increasing the risk of post-operative complication and leakage. Moreover, most of the fistulas will close on their own. In young healthy patients who have a higher cumulative risk of developing secondary complications from persistent bilioenteric fistulas, one-stage surgery may be indicated [20]. The treatment approach should be selected based on each patient's age, comorbidities, and the availability of endoscopists/surgeons.

## Conclusions

In summary, we presented the case of an elderly man with Bouveret’s syndrome who had experienced a favorable outcome after endoscopic treatment alone using holmium:YAG laser for gallstone fragmentation. Although holmium:YAG laser lithotripsy is not a common endoscopic modality in gastroenterology, this case report shows that holmium:YAG laser is a promising tool for Bouveret’s syndrome. Holmium:YAG laser lithotripsy is capable of delivering a favorable outcome in a complicated patient, as seen in this present case with a heavily calcified and pigmented stone, older age, and multiple comorbidities. Holmium:YAG laser lithotripsy could be considered for use with endoscopic equipment for the future management of this condition in patients who have a high risk for surgery and heavily calcifies gallstones.
